# Supplementary material for: Detection of Streptococcus pyogenes M1UK in Australia and characterization of the mutation driving enhanced expression of superantigen SpeA
Source: Nat Commun. 2023 Feb 24;14:1051. doi: 10.1038/s41467-023-36717-4 (PMC9951164; doi:10.1038/s41467-023-36717-4)
Supplement: Supplementary file 1 — Supplementary Information [file 41467_2023_36717_MOESM1_ESM.pdf]

Supplementary Information

Detection of *Streptococcus pyogenes* $M1_{UK}$ in Australia and characterization of the mutation driving enhanced expression of superantigen SpeA

Davies *et al.*

Supplementary Figures

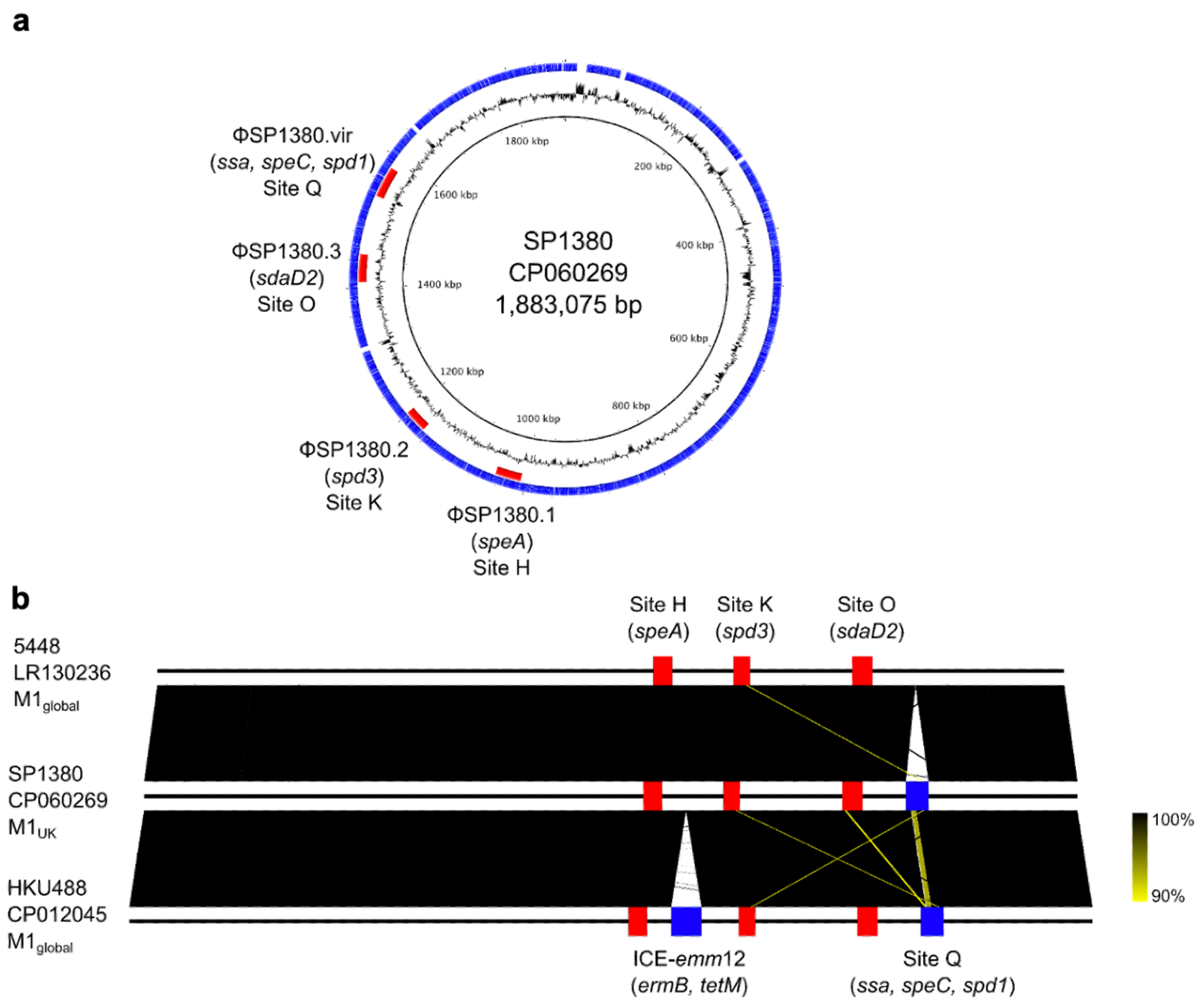

Supplementary Figure 1. Genomic features of the Australian M1$_{UK}$ strain SP1380. (**a**) Genome ring of the 1,833,075 bp SP1380 genome showing GC plot, position of prophage elements (red blocks), and location of coding sequences (blue). Name, associated virulence gene and chromosomal integration site of prophage elements[1] are annotated. (**b**) Comparative BlastN alignment of 3 M1 *S. pyogenes* complete genome sequences 5448 (M1$_{global}$); SP1380 (M1$_{UK}$) and HKU488 (M1$_{global}$) with location of mobile genetic elements and associated chromosomal integration site annotated. In blue are the elements not shared by all 3 strains. GenBank accession numbers are indicated under strain name. Pairwise nucleotide sequence identity is shown (key represents the BlastN percentage). The Australian M1$_{UK}$ strain SP1380

has acquired a prophage carrying the virulence repertoire *ssa*, *speC* and *spd1* (termed ϕSP1380.vir) similar to that carried by the scarlet fever outbreak strain from Hong Kong ϕHKU488.vir (refer to Figure 1c).

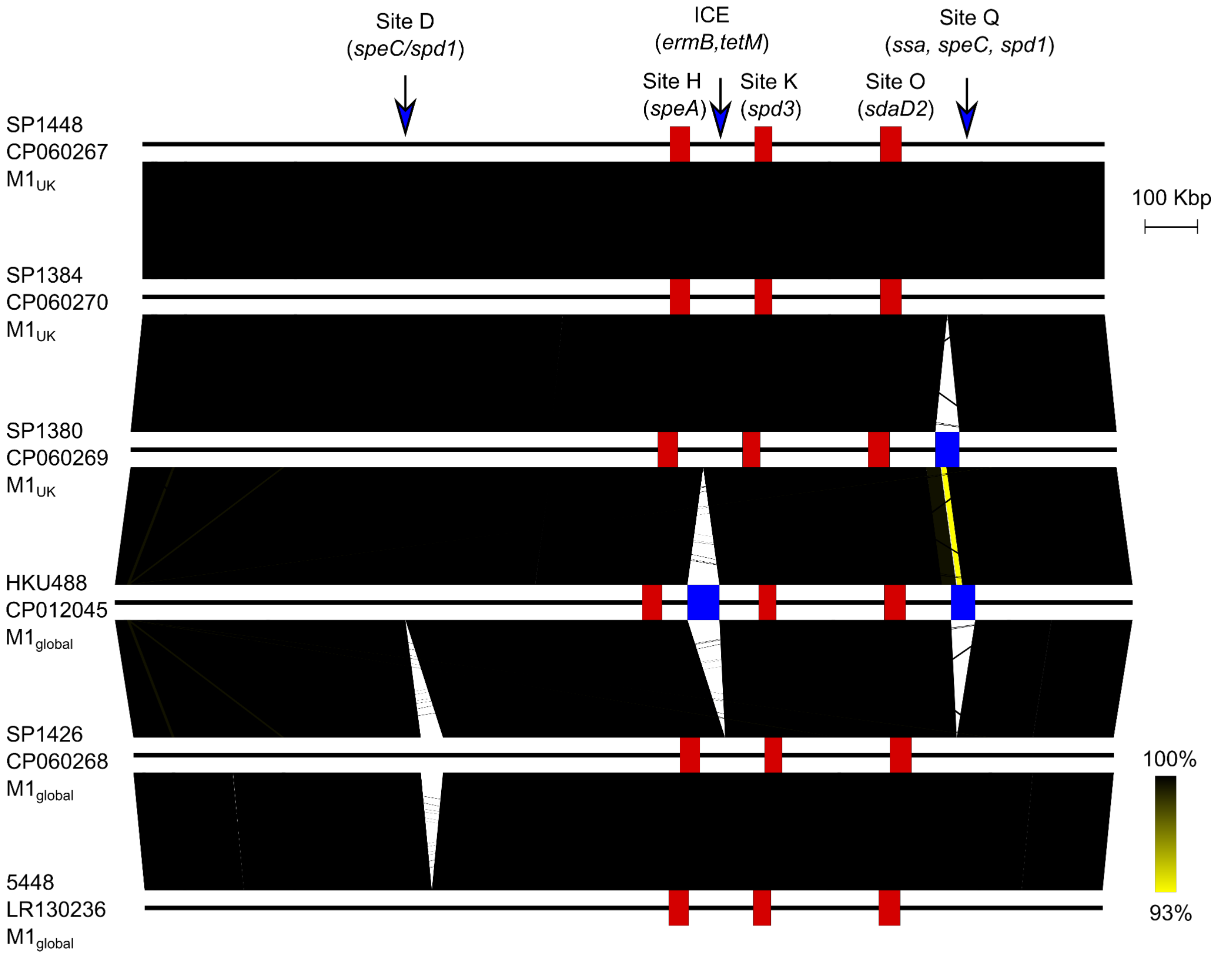

Supplementary Figure 2. Whole genome alignment of new Australian M1$_{global}$ (SP1426) and M1$_{UK}$ (SP1448, SP1380, SP1384) genome sequences to reference genomes from other geographical locations (5448, USA, M1$_{global}$; HKU488, Hong Kong, M1$_{global}$). Coloured blocks dictate location of mobile genetic elements (red, refer to elements common across the 6 reference genomes; blue, variably carried elements). Key represents nucleotide homology. Relative position of prophage chromosomal ‘sites’[1] and Integrative Conjugative Element [ICE] location and their associated virulence or antimicrobial resistance genes) are annotated above the genome alignments. GenBank accession numbers and associated M1 genotype for each reference genome is provided under each strain name. Figure was generated using EasyFig v2.2.2.

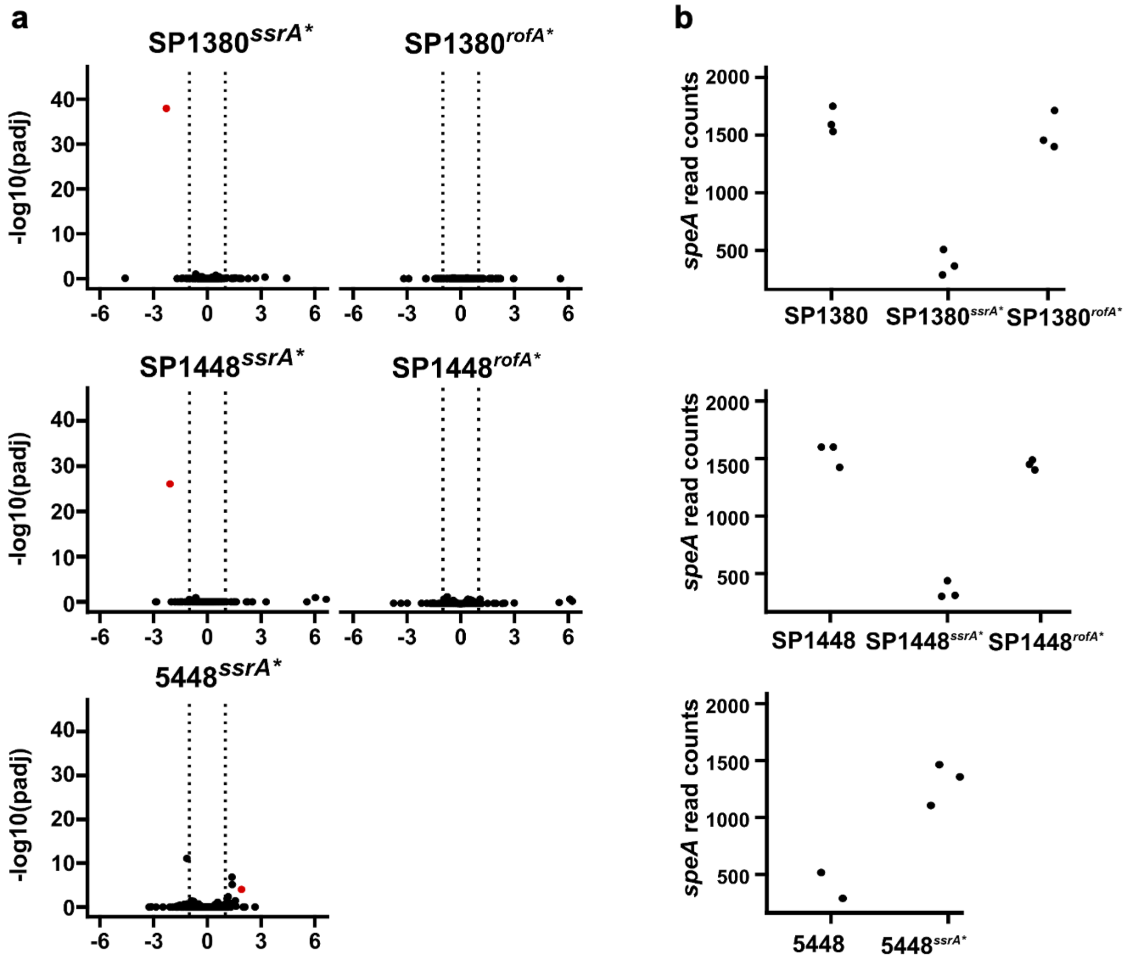

Supplementary Figure 3. Differential gene expression profile of Australian M1UK strains SP1380 and SP1448 *ssrA* and *rofA* isogenic mutants and the M1global strain 5448 *ssrA* mutant. For M1UK genotypes (SP1380 and SP1448), isogenic mutants refer to reversion of *ssrA* and *rofA* SNPs to M1global-like, while 5448^{ssrA*} refers to +5G>C mutation in the 5'-leader sequence of the *ssrA* gene (M1UK-like). All experiments represent biological triplicates with the exception of 5448 which represents a biological duplicate due to sequence contamination. (**a**) Volcano plot of differentially expressed genes from mid log-phase cultures relative to parental wildtype strains, determined using a two-sided Wald tests with Benjamini Hochberg correction for multiple comparison from the DESeq2 package. X-axis refers to $\log_2$ of relative fold-change and y-axis indicates $\log_{10}$ Benjamini-Hochberg adjusted p-value. Red dot refers to differential expression of the *speA* gene. Dotted lines refer to cut-off's applied for differential relative fold change (≥2 fold change). (**b**) Normalized *speA* read counts of Australian M1UK

strains SP1380, SP1448 and *ssrA* and *rofA* isogenic mutants and the $M1_{global}$ strain 5448 and isogenic mutant 5448^{ssrA*}. In all three genetic backgrounds, alteration of a single SNP (+5G>C) in the 5'-leader sequence of *ssrA* altered *speA* gene expression.

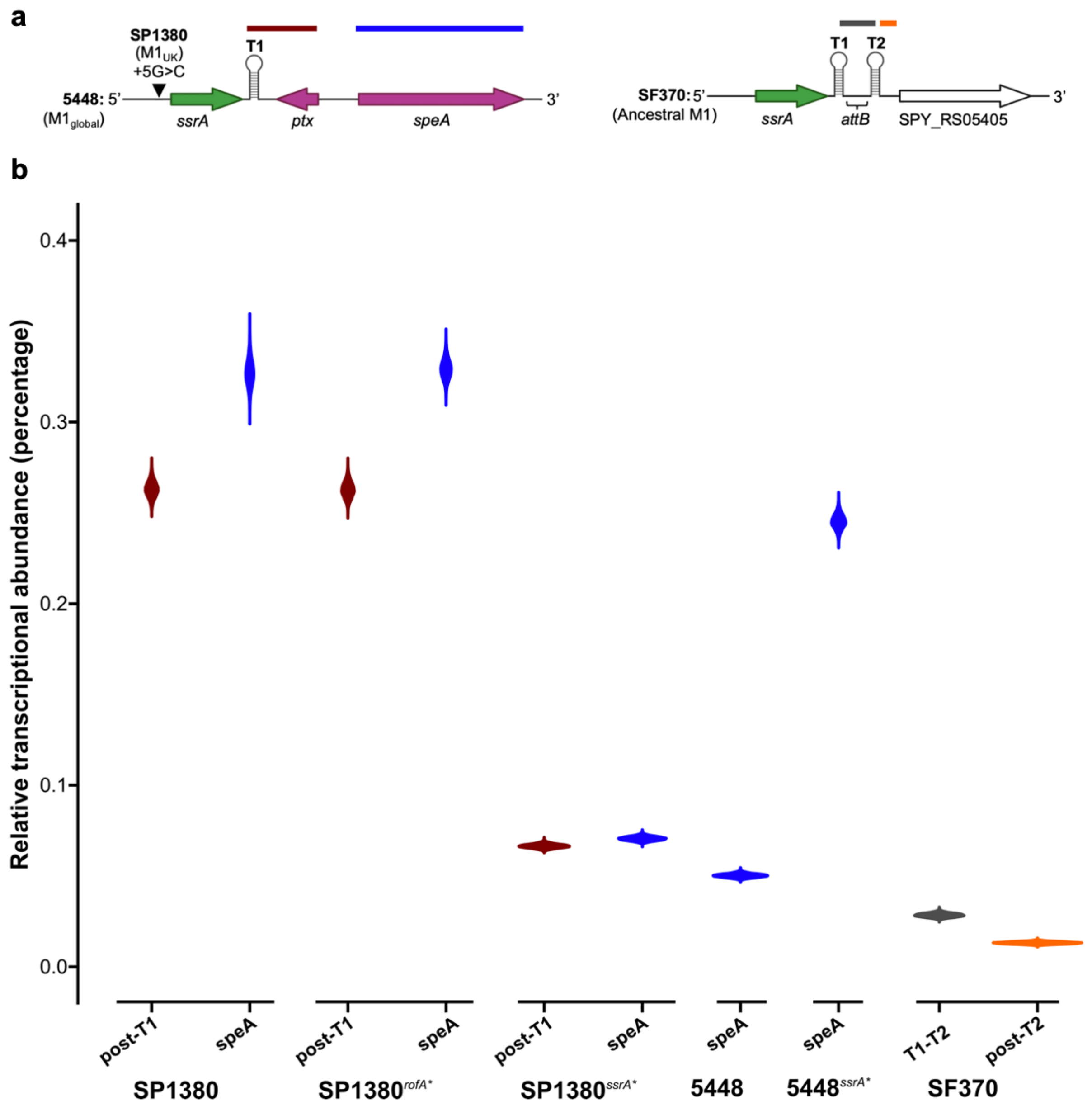

Supplementary Figure 4. Variation in the level of *ssrA* transcriptional read-through across different M1 *S. pyogenes* genetic backgrounds and associated isogenic mutants. (**a**) Schematic representation of *ssrA* genomic region within $M1_{global}$ (5448), $M1_{UK}$ (SP1380) and SF370 (ancestral M1 genotype - lacking *speA*-containing prophage). Two predicted Rho-independent transcriptional terminators of *ssrA* are annotated (T1 and T2) with the location of T2 disrupted by presence of *speA* prophage in modern $M1_{global}$ and $M1_{UK}$ genotypes. Coloured bars above each schematic refer to regions used to define transcriptional read depth coverage in (**b**). (**b**) Violin plot of relative *ssrA* transcriptional read-through in different M1 genomic

backgrounds and isogenic mutants. Data is derived from Illumina RNAseq data. *ssrA* transcriptional read-through is defined as mean read coverage at genomic regions immediately downstream of proposed *ssrA* transcriptional terminators. Genomic regions are defined per genome: SP1380 post-T1 to region before low G/C region [refer to Figure 3a] (1,006,939 - 1,007,531 bp), SP1380 *speA* open reading frame (1,007,498 - 1,008,253 bp), SP1380 *ssrA* open reading frame (1,006,592 - 1,006,938 bp); 5448 *ssrA* open reading frame (855,001 - 855,348 bp); 5448 *speA* open reading frame (853,686 - 854,441 bp); SF370 between terminators T1-T2 (1,065,434 - 1,065,588 bp); SF370 post-T2 to downstream gene promoter (1,065,589 - 1,065,674 bp); SF370 *ssrA* open reading frame (1,065,025 - 1,065,372 bp). A read-through distribution is determined by mean coverage of genomic regions, normalized to read coverage of *ssrA* open reading frame, sampled randomly 10,000 times. These data indicate a 4 to 5-fold difference in relative transcriptional read-through past the *ssrA*-T1 and into the *speA* gene in M1 *S. pyogenes* strains containing the +5G-C SNP in the leader of *ssrA* (a signature of the $M1_{UK}$ genotype).

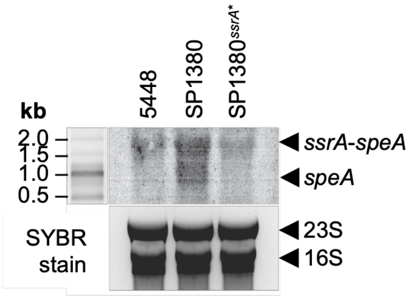

Supplementary Figure 5. Northern blot of total RNA from M1 *S. pyogenes* strains probed for *speA* defined using a 43-mer anti-sense oligonucleotide (*Supplementary Table 3*). Arrows indicate possible mono- (~0.8 kb) and bicistronic (~1.8 kb) *speA* transcripts. SYBR stained 23S and 16S rRNA were used as an RNA loading control.

Supplementary Tables

Supplementary Table 1. List of molecular markers that differentiate the M1$_{UK}$ genotype compared to the ancestral M1$_{global}$ genotype.

Position	Locus Tag	Gene	Protein Change	Product	Ref	Alt
76208$	Spy0065	*adk*	Glu151	adenylate kinase	AAAG	A
115646#	Spy0106	*rofA*	Asp491Asn	transcriptional regulator	C	T
116162+#	Spy0106	*rofA*	MetPhe318 IleVal	transcriptional regulator	AC	CA
250832#	Spy0243	-	Asn248Asn	ABC transporter-associated protein	T	C
343279$	intergenic	-	-	exoA, lctO	TA	T
513254	Spy0525	-	Ala116Ser	galactose-6-phosphate-Isomerase lacB subunit	G	T
528360#	intergenic	*asnS*	-	ATP-binding protein	A	T
563631#	Spy0566	*sagE*	Ala52Thr	streptolysin synthesis	G	A
613633	Spy0609	*gacH*	Leu300Pro	phosphoglycerol transferase	T	C
626494	Spy0623	-	Leu35Leu	methyltransferase	G	A
661707#	Spy0656	*trmD*	Ala44Thr	tRNA (guanine-N(1)-)-methyltransferase	G	A
730823	Spy0727	*recJ*	Ala336Val	single-stranded-DNA-specific exonuclease	C	T
784467#	Spy0779	-	Val147Val	putative membrane spanning protein	T	C
819098#	Spy0825	*murB*	Ala82Thr	UDP-N-acetylenolpyruvoylglucosamine reductase	G	A

923079[#]	Spy0933	-	Ala101Val	putative NADH-dependent flavin oxidoreductase	G	A
942633[#]	Spy0951	*pstB*	His123Asn	phosphate transport ATP-binding protein	G	T
983438[#]	intergenic	-	-	5' transcriptional leader of *ssrA* (transfer-messenger RNA)	G	C
1082253	Spy1108	*metK2*	Ala221Thr	S-adenosylmethionine synthetase	C	T
1238124	Spy1282	*msrA*	Ala32Val	peptide methionine sulfoxide reductase	G	A
1238673	Spy1283	*tlpA*	Ala71Val	thiol:disulfide interchange protein	G	A
1251193[#]	Spy1293	-	Ser135Leu	hypothetical protein	G	A
1294671[$]	intergenic	-	-	comFA, Xaa-Pro dipeptidase	CT	C
1373176[#]	Spy1400	-	Met66Ile	PTS system, galactose-specific IIB component	C	A
1407497	Spy1439	-	Gly290Glu	portal protein	C	T
1446116	Spy1490	-	Thr231Thr	3-oxoacyl-[acyl-carrier protein] reductase	C	T
1535209[#]	intergenic	-	-	upstream of *glA* (*glpF.2*) putative glycerol uptake facilitator protein, universal stress protein	A	G
1630160[#$]	intergenic	*polC*	-	*polC, proS*	CT	C
1702540[#]	Spy1741	*gldA*	Trp175*	glycerol dehydrogenase	C	T
1734749	Spy1772	-	Ala61Thr	glutamate formiminotransferase	G	A
1828734[#]	Spy1860	-	Gly71Arg	putative membrane spanning protein	G	A

Footnotes:

Position, Locus Tag, Product annotations are relative to MGAS5005 reference genome (GenBank CP000017.2)

Molecular events associated with the evolutionary intermediate M1 population ($M1_{inter}$)

+ Defined as 2 separate SNPs by Lynskey *et al*[2].

Acronyms: Ref, Reference; Alt, Alternate

$ additional small deletion events identified in this study that are $M1_{UK}$ lineage defining

* Refers to a SNP resulting in a frameshift in the *gldA* gene resulting in a premature stop codon.

Supplementary Table 2. Social distancing measures introduced in Queensland across 2020 and 2021 to combat the COVID-19 pandemic effectively suppressed other respiratory infections such as pertussis and influenza[3], but with relatively less impact on scarlet fever and *S. pyogenes* invasive disease case numbers. While useful for monitoring trends, this hospital emergency department admission data will only account for a relatively small proportion of cases such as scarlet fever, as most patients are likely to be managed within primary care settings.

	Yearly Hospital Presentations				
**Year**	2017	2018	2019	2020	2021
**Pertussis**	1343	1763	1765	490	99
**Influenza**	56616	15705	68151	6047	296
**Scarlet fever**	480	445	429	296	388
**invasive** *S. pyogenes*	381	354	340	279	279

Supplementary Table 3. Bacterial strains, plasmids and primers used for experimental analyses in this study.

Bacterial strains	Description	Reference/ Source
E. coli		
MC1061	Laboratory cloning strain	[4]
TOP10	Laboratory cloning strain	Invitrogen
S. pyogenes		
SF370	Ancestral M1 strain	[5]
5448	Invasive M1$_{global}$ isolate	[6]
HKU488	Scarlet feverM1$_{global}$ isolate (Hong Kong)	[7]
SP1380	Scarlet fever M1$_{UK}$ isolate (Australia)	This study
SP1384	Scarlet fever M1$_{UK}$ isolate (Australia)	This study
SP1426	Scarlet fever M1$_{global}$ isolate (Australia)	This study
SP1448	Invasive M1$_{UK}$ isolate (Australia)	This study
5448^{ssrA*}	5448 isogenic mutant strain containing the M1$_{UK}$ intergenic G to C (position 983438$^{\#}$) SNP upstream of *ssrA*	This study
5448$^{T1\text{-}GC>CG}$	5448 isogenic mutant strain containing GC to CG (positions 983845 and 983852$^{\#}$) SNPs in the T1 terminator stem structure	This study
SP1380^{rofA*}	SP1380 isogenic mutant strain containing three *rofA* repaired (M1$_{global}$-like) SNPs T to C (position 115646$^{\#}$) and CA to AC (position 116162$^{\#}$)	This study
SP1380^{ssrA*}	SP1380 isogenic mutant strain containing the repaired (M1$_{global}$-like) intergenic C to G (position 983438$^{\#}$) SNP upstream of *ssrA*	This study
SP1380^{T2}	SP1380 isogenic mutant strain containing the T2 terminator sequence from SF370 between *ssrA* and *speA* (SF370-like)	This study

SP1448^{rofA*}	SP1448 isogenic mutant strain containing three *rofA* repaired ($M1_{global}$-like) SNPs T to C (position 115646#) and CA to AC (position 116162#)	This study
SP1448^{ssrA*}	SP1448 isogenic mutant strain containing the repaired ($M1_{global}$-like) intergenic C to G (position 983438#) SNP upstream of *ssrA*	This study
**Plasmids**		
pLZts	Temperature-sensitive shuttle plasmid, spectinomycin^R	8
pLZts-*ssrA**_$M1_{UK}$	pLZts construct containing the $M1_{UK}$ intergenic G to C (position 983438#) SNP upstream of *ssrA*	This study
pLZts-*ssrA**_$M1_{global}$	pLZts construct containing the repaired ($M1_{global}$-like) intergenic C to G (position 983438#) SNP upstream of *ssrA*	This study
pLZts-*rofA**_$M1_{global}$	pLZts construct containing three *rofA* repaired ($M1_{global}$-like) SNPs T to C (position 115646#) and CA to AC (position 116162#)	This study
pLZts-*ssrA*T2_M1	pLZts construct containing the T2 terminator sequence from SF370 between *ssrA* and *speA* (SF370-like)	This study
pLZts-*ssrA*T1_GC>CG	pLZts construct containing GC to CG (positions 983845 and 983852#) SNPs in the T1 terminator stem structure	This study

Primers	Sequence 5’-3’
Primers for cloning	
SNPs_*rofA*_F	GTCGTCAGACTGATGGGCCCTTCTTTAAATTAAAGCAATAAACTTG
SNPs_*rofA*_R	CATAACCTGAAGGAAGATCTGCTCTGATTCGGTTAAGTAG
SNP_*ssrA*_F	TGATGGGCCCTCTCCAAACGCCTTTCATAC
SNP_*ssrA*_R	AGGAAGATCTCACCAATATTACTAACAATGAAAAAATAG
5’$M1_{UK}$_*ssrA*T1_F	GTCGTCAGACTGATGGGCCCGTACCAATTAGTGCAATAATTC
5’$M1_{UK}$_*ssrA*T1_R	GGATAACGGAAACTCATGAACAAGACAAAAAG
M1_*ssrA*T2_F	TTCATGAGTTTCCGTTATCCCTATAGCC
M1_*ssrA*T2_R	AAGTTATGGTATTATAAACAAGATATAGCTATGATTC

3’M1$_{UK}$_*ssrA*T1_F	TGTTTATAATACCATAACTTTCTATATTATTGACAAC
3’M1$_{UK}$_*ssrA*T1_R	CATAACCTGAAGGAAGATCTCATTCTCGTGAGTAACAG
*ssrA*T1_GC>CG_F	CAAGACAAAAAGAAAAAACCTTCATGTAAGAAGGTTTTA GTAAGTTATGATTACTTACGG
*ssrA*T1_GC>CG_R	CCGTAAGTAATCATAACTTACTAAAACCTTCTTACATGAA GGTTTTTTCTTTTTGTCTTG
Primers for qPCR	
qPCR-*proS*-F	AGCTGATCTCTGGCGTGAAT
qPCR-*proS*-R	CGCACCAAAGTCGTAAAGGT
qPCR-*speA*-F	TGTTTCAGGGCCAAATTATGA
qPCR-*speA*-R	CATGCACTCCTTTCTGCATT
Oligonucleotide for Northern blotting	
S.pyogenes.SpeA.2. NB	AGGAATTTCTAAATGATTCCCTTCATGATTTGTTACCCCTC CG

Footnote:

#Position is relative to MGAS5005 reference genome (GenBank CP000017.2).

Supplementary Table 4. Differential gene expression values (*p*>0.05, ≥2 fold-change) of 5448^{ssrA*} relative to wildtype 5448.

locus_tag	names	product	baseMean read counts$^{\&}$	Log2 Fold-Change	pvalue	padj
RS01605	NA	MFS transporter	52746	-1.147358555	3.15E-15	5.38E-12
RS04435	*speA*	streptococcal pyrogenic exotoxin	943	1.902527071	2.31E-06	0.00098378
RS08015	*lacG*	6-phospho-beta-galactosidase	2224	1.381231493	9.62E-08	5.47E-05
RS08020	*lacE*	PTS transporter subunit EIIC	3914	1.368558547	1.37E-09	1.17E-06
RS08030	*lacD.2*	tagatose-bisphosphate aldolase	2625	1.156170295	4.01E-05	0.01367717
RS08035	*lacC.2*	tagatose-6-phosphate kinase	2173	1.098204169	0.00011919	0.03389034

Footnotes:

$^{\&}$ Normalized read-count value provided

NA, no gene name currently assigned.

References

1. McShan, W. M., McCullor, K. A. & Nguyen, S. V. The bacteriophages of *Streptococcus pyogenes. Microbiol. Spectr.* 7, doi: 10.1128/microbiolspec.GPP3-0059-2018 (2019).
2. Lynskey, N. N. et al. Emergence of dominant toxigenic M1T1 *Streptococcus pyogenes* clone during increased scarlet fever activity in England: a population-based molecular epidemiological study. *Lancet Infect. Dis.* 19, 1209–1218 (2019).
3. The State of Queensland, Queensland Health, Communicable Diseases Branch, Prevention Division. Notifiable conditions annual reporting. https://www.health.qld.gov.au/clinical-practice/guidelines-procedures/diseases-infection/surveillance/reports/notifiable/annual.
4. Wertman, K. F., Wyman, A. R. & Botstein, D. Host/vector interactions which affect the viability of recombinant phage lambda clones. *Gene* 49, 253–262 (1986).
5. Ferretti, J. J. et al. Complete genome sequence of an M1 strain of *Streptococcus pyogenes*. *Proc. Natl. Acad. Sci. USA* 98, 4658–4663 (2001).
6. Chatellier, S. et al. Genetic relatedness and superantigen expression in group A *Streptococcus* serotype M1 isolates from patients with severe and nonsevere invasive diseases. *Infect. Immun.* 68, 3523–3534 (2000).
7. Zakour, N. L. B. et al. Transfer of scarlet fever-associated elements into the group A *Streptococcus* M1T1 clone. *Sci. Rep.* 5, 15877 (2015).
8. Barnett, T. C., Daw, J. N., Walker, M. J. & Brouwer, S. Genetic manipulation of group A *Streptococcus* — gene deletion by allelic replacement. in group A *Streptococcus*: Methods and Protocols (eds. Proft, T. & Loh, J. M. S.) 59–69 (Springer US, 2020).
