# Supplementary material for: Detection of Streptococcus pyogenes M1UK in Australia and characterization of the mutation driving enhanced expression of superantigen SpeA
Source: Nat Commun. 2023 Feb 24;14:1051. doi: 10.1038/s41467-023-36717-4 (PMC9951164; doi:10.1038/s41467-023-36717-4)
Supplement: Supplementary file 3 — Description of Additional Supplementary Files [file 41467_2023_36717_MOESM3_ESM.pdf]

Description of Additional Supplementary Files

File Name: Supplementary Data 1
Description: Metadata and genome accession numbers of M1 GAS genome sequences used in this study
