# Supplementary material for: Detection of Streptococcus pyogenes M1UK in Australia and characterization of the mutation driving enhanced expression of superantigen SpeA
Source: Nat Commun. 2023 Feb 24;14:1051. doi: 10.1038/s41467-023-36717-4 (PMC9951164; doi:10.1038/s41467-023-36717-4)
Supplement: Supplementary file 5 — Reporting Summary [file 41467_2023_36717_MOESM5_ESM.pdf]

nature portfolio

Corresponding author(s): Mark J Walker

Last updated by author(s): Feb 2, 2023

Reporting Summary

Nature Portfolio wishes to improve the reproducibility of the work that we publish. This form provides structure for consistency and transparency in reporting. For further information on Nature Portfolio policies, see our Editorial Policies and the Editorial Policy Checklist.

Statistics

For all statistical analyses, confirm that the following items are present in the figure legend, table legend, main text, or Methods section.

n/a	Confirmed	
☐	☒	The exact sample size (*n*) for each experimental group/condition, given as a discrete number and unit of measurement
☐	☒	A statement on whether measurements were taken from distinct samples or whether the same sample was measured repeatedly
☐	☒	The statistical test(s) used AND whether they are one- or two-sided<br>*Only common tests should be described solely by name; describe more complex techniques in the Methods section.*
☒	☐	A description of all covariates tested
☐	☒	A description of any assumptions or corrections, such as tests of normality and adjustment for multiple comparisons
☐	☒	A full description of the statistical parameters including central tendency (e.g. means) or other basic estimates (e.g. regression coefficient) AND variation (e.g. standard deviation) or associated estimates of uncertainty (e.g. confidence intervals)
☐	☒	For null hypothesis testing, the test statistic (e.g. *F*, *t*, *r*) with confidence intervals, effect sizes, degrees of freedom and *P* value noted<br>*Give P values as exact values whenever suitable.*
☒	☐	For Bayesian analysis, information on the choice of priors and Markov chain Monte Carlo settings
☒	☐	For hierarchical and complex designs, identification of the appropriate level for tests and full reporting of outcomes
☒	☐	Estimates of effect sizes (e.g. Cohen's *d*, Pearson's *r*), indicating how they were calculated

Our web collection on statistics for biologists contains articles on many of the points above.

Software and code

Policy information about availability of computer code

Data collection	No software was used for data collection
Data analysis	Data analysis was performed using the following software:<br>Trimmomatic v0.39 (https://github.com/timflutre/trimmomatic)<br>Kraken (v0.10.5-beta, https://github.com/DerrickWood/kraken)<br>shovill v1.0.9 (https://github.com/tseemann/shovill)<br>Spades v3.13.0 assembler<br>prokka v1.14.0 annotator<br>Guppy v3.0.6<br>Unicycler v0.4.7<br>filtlong v0.2.0<br>PGAP v4.12 (National Center for Biotechnology Information (NCBI))<br>MAUVE genome aligner v2.4.0<br>ekidna v0.3.0 (https://github.com/tseemann/ekidna)<br>paftools (minimap2 v2.18)<br>vcf-isec (VCFtools) v0.1.16<br>BWA-MEM2, snippy v4.6.0 (github/tseemann/snippy)<br>SnpEff v4.3t<br>Gubbins v2.4.0<br>IQ-TREE v1.6.12<br>ggtree v2.0.1

tidyverse v1.3.0
phangorn v2.5.5
treeio v1.10.0
phytools v0.6-99
screen_assembly v1.2.7
emm-typer commit: 500d048 on branch: master (https://github.com/MDU-PHL/emmtyper)
tblastN, Easyfig v2.2.2
FastQC v0.11.0
MultiQC v1.9
TrimGalore v0.6.5
SortMeRNA v4.2.0
BWA-MEM v0.7.17
featureCounts, Subreads v2.0.0
DEseq2 v1.32.0 (R 4.1.1)
edgeR v2.23.1 (R 4.1.1)
bamCoverage (Deeptools v3.5.0)
ggplot2 v3.3.5
Guppy v5.0.17 (High-accuracy model, min_qscore 7)
SeqKit v2.2.0
cutadapt v3.8.6
QuantStudio Real-Time PCR software v1.1 (QuantStudio 6 Flex, Life Technologies)
Prism v9.4.1(GraphPad)

For manuscripts utilizing custom algorithms or software that are central to the research but not yet described in published literature, software must be made available to editors and reviewers. We strongly encourage code deposition in a community repository (e.g. GitHub). See the Nature Portfolio guidelines for submitting code & software for further information.

Data

Policy information about availability of data

All manuscripts must include a data availability statement. This statement should provide the following information, where applicable:

- Accession codes, unique identifiers, or web links for publicly available datasets
- A description of any restrictions on data availability
- For clinical datasets or third party data, please ensure that the statement adheres to our policy

Data Availability
The complete annotated genome sequences generated in this study have been deposited in the NCBI database under the BioProject PRJNA656382 (https://www.ncbi.nlm.nih.gov/bioproject/?term=PRJNA656382) with the GenBank accession numbers CP060267 (SP1448), CP060268 (SP1426), CP060269 (SP1380) and CP060270 (SP1384). Illumina short-reads of 318 M1 S. pyogenes from Australia have been deposited under the BioProject PRJNA872282 (https://www.ncbi.nlm.nih.gov/bioproject/?term=PRJNA872282). The RNA-seq reads and associated gene expression profiles have been deposited in NCBI's Gene Expression Omnibus under the SuperSeries accession number GSE212243 (https://www.ncbi.nlm.nih.gov/geo/query/acc.cgi?acc=GSE212243). The SP1380 ONT direct RNA reads are available in the NCBI repository BioProject PRJNA872764 (SRR21185202) (https://www.ncbi.nlm.nih.gov/bioproject/?term=PRJNA872764).
Source data are provided with this paper.

Human research participants

Policy information about studies involving human research participants and Sex and Gender in Research.

Reporting on sex and gender	not applicable
Population characteristics	not applicable
Recruitment	not applicable
Ethics oversight	not applicable

Note that full information on the approval of the study protocol must also be provided in the manuscript.

Field-specific reporting

Please select the one below that is the best fit for your research. If you are not sure, read the appropriate sections before making your selection.

☒ Life sciences ☐ Behavioural & social sciences ☐ Ecological, evolutionary & environmental sciences

For a reference copy of the document with all sections, see nature.com/documents/nr-reporting-summary-flat.pdf

Life sciences study design

All studies must disclose on these points even when the disclosure is negative.

Sample size	Sample sizes were not predetermined based on statistical methods, but were chosen according to the standards in the field (e.g. PMID: 25401300, PMID: 33024089) - at least 3 independent biological replicates for each condition. This generated sufficient data for statistical analysis.
Data exclusions	No data were excluded from the analyses
Replication	Numbers of experimental replicates are stated in each figure legend. Reported results were consistently replicated across multiple experiments with all replicates generating similar results.
Randomization	No randomization was necessary as experiments were performed with appropriate controls. Randomization is not generally used in this field.
Blinding	Investigators were not blinded. Blinding during analysis was not necessary because the results are quantitative and did not require subjective judgment or interpretation. Blinding is not typically used in the field. Additionally, blinding was not possible since each experiment was conducted by a single person and sample labeling was required.

Reporting for specific materials, systems and methods

We require information from authors about some types of materials, experimental systems and methods used in many studies. Here, indicate whether each material, system or method listed is relevant to your study. If you are not sure if a list item applies to your research, read the appropriate section before selecting a response.

Materials & experimental systems

n/a	Involved in the study	
☐	☒	Antibodies
☒	☐	Eukaryotic cell lines
☒	☐	Palaeontology and archaeology
☒	☐	Animals and other organisms
☒	☐	Clinical data
☒	☐	Dual use research of concern

Methods

n/a	Involved in the study	
☒	☐	ChIP-seq
☒	☐	Flow cytometry
☒	☐	MRI-based neuroimaging

Antibodies

Antibodies used	Affinity-purified rabbit antibody to SpeA (PAI111, Toxin Technology; 1:1000 dilution), affinity-purified rabbit antibody to SpeC (PCI333, Toxin Technology; 1:1000 dilution), affinity-purified rabbit antibody to SSA (produced by Mimotopes, Clayton, Australia raised against the peptide HCGGSSQPDPTPEQLNKSSQFTG-OH coupled to Keyhole Limpet Hemocyanin; 1:500 dilution). Mouse antibody to Spd1 (1:1000 dilution) were generated as previously described (PMID: 33024089). Anti-rabbit IgG (H+L) (DyLight 800 4X PEG Conjugate, NEB, 5151P) and anti-mouse IgG (H+L) (DyLight 800 4× PEG Conjugate, NEB, 5257S) were used as the secondary antibodies (1:10,000 dilution).
Validation	Specificity of each primary antibody was validated by Western Blotting using purified recombinant protein and culture supernatants of respective isogenic mutant strains of Streptococcus pyogenes. Detection with secondary antibodies validated the species-specific source of each primary antibody.
